# The roles of Tenascin C and Fibronectin 1 in adhesive capsulitis: a pilot gene expression study

**DOI:** 10.6061/clinics/2016(06)07

**Published:** 2016-06

**Authors:** Carina Cohen, Mariana Ferreira Leal, Paulo Santoro Belangero, Eduardo Antônio Figueiredo, Marília Cardoso Smith, Carlos Vicente Andreoli, Alberto de Castro Pochini, Moises Cohen, Benno Ejnisman, Flávio Faloppa

**Affiliations:** I Universidade Federal de São Paulo, Departamento de Ortopedia e Traumatologia; II Departamento de Morfologia e Genética, Disciplina de Genética, São Paulo/SP, Brazil

**Keywords:** Adhesive Capsulitis, Glenohumeral Capsule, Gene Expression, Extracellular Matrix, *TGFβ1* Signaling

## Abstract

**OBJECTIVES:**

We evaluated mRNA expression levels of genes that encode TGF-β1; the TGF-β1 receptor; the collagen-modifying enzymes LOX, PLOD1, and PLOD2; and the extracellular matrix proteins COMP, FN1, TNC and TNXB in synovial/capsule specimens from patients with idiopathic adhesive capsulitis. Possible associations between the measured mRNA levels and clinical parameters were also investigated.

**METHODS:**

We obtained glenohumeral joint synovium/capsule specimens from 9 patients with idiopathic adhesive capsulitis who had not shown improvement in symptoms after 5 months of physiotherapy. Adhesive capsulitis was confirmed in all patients by magnetic resonance imaging. We also obtained specimens from 8 control patients who had underwent surgery for acute acromioclavicular joint dislocation and who had radiological indication of glenohumeral capsule alteration based on arthroscopic evaluation. mRNA expression in the synovium/capsule specimens was analyzed by quantitative reverse transcription PCR. The *B2M* and *HPRT1* genes were used as references to normalize target gene expression in the shoulder tissue samples.

**RESULTS:**

The synovium/capsule samples from the patients with adhesive capsulitis had significantly higher *TNC* and *FN1* expression than those from the controls. Additionally, symptom duration directly correlated with expression of *TGFβ1 receptor I*.

**CONCLUSION:**

Elevated levels of *TNC* and *FN1* expression may be a marker of capsule injury. Upregulation of *TGFβ1 receptor I* seems to be dependent on symptom duration; therefore, *TGFβ* signaling may be involved in adhesive capsulitis. As such, *TNC, FN1* and *TGFβ1 receptor I* may also play roles in adhesive capsulitis by contributing to capsule inflammation and fibrosis.

## INTRODUCTION

Adhesive capsulitis, or frozen shoulder, is a debilitating condition in which patients present limited active and passive glenohumeral motion. Adhesive capsulitis occurs in 3%-5% of the general population 1 and the main cause of the painful restriction of movement is inflammatory contracture of the joint capsule. The initial inflammation seems to lead to capsular fibrosis, stiffness and pain 2. Therefore, it has been hypothesized that similarities exist between adhesive capsulitis and the fibrous contractures that occur in Dupuytren disease 3,4. However, the molecular mechanism responsible for the underlying glenohumeral capsule inflammation and fibrosis is poorly understood.

Rodeo et al. suggested that cytokines, such as transforming growth factor beta (TGFβ), may be involved in the inflammatory and fibrotic processes that occur in adhesive capsulitis. These cytokines may cause abnormal regulation of collagen expression and augment fibroblast proliferation 5. Therefore, TGFβ acts as a persistent stimulus that leads to capsular fibrosis.

TGFβ induces fibroblasts to synthesize, remodel and contract extracellular matrix (ECM), making this cytokine a key mediator of the fibrotic response 6. TGFβ is activated by proteolytic cleavage 7 mediated by the signaling receptors TGFβ receptor I (TGFβR1) and TGFβ receptor 2 (TGFβR2) 8. TGFBR1 is the principal propagator of TGFβ signaling 9.

TGFβ1, a key member of the TGFβ superfamily, regulates the collagen-modifying enzymes lysyl oxidase (LOX) 10 and lysyl hydroxylases 1 and 2 (encoded by the *PLOD1* and *PLOD2* genes, respectively) 11-13. LOX plays a role in connective tissue matrix biogenesis through the oxidation of lysine residues in collagen and elastin, contributing to the formation of covalent cross-links and thereby stabilizing fibrous ECM proteins 14,15. In several fibrotic injuries, TGFβ1 controls the expression and enzymatic activity of LOX 10.

Lysyl hydroxylases such as PLOD1 and PLOD2 promote cross-linking in ECM molecules, which contribute to ECM structural stability and maturation 16,17. Increased *PLOD2* expression has been reported in fibroblasts isolated from hypertrophic scars, keloids and *palmar fascia* from patients with Dupuytren disease 18. To the best of our knowledge, no previous studies have evaluated the roles of lysyl oxidase and hydroxylase in adhesive capsulitis.

TGFβ regulates and is regulated by several ECM proteins. Cartilage oligomeric matrix protein (COMP), a glycoprotein found in the ECM of joints, plays a catalytic role in fibrillogenesis 19. Recent studies have shown that COMP also directly binds members of the TGFβ superfamily of proteins, including TGFβ1 20. Haudenschild et al. showed that TGFβ1 displays enhanced bioactivity when bound to COMP 20. In addition, TGFβ1 appears to have the capacity to induce COMP expression 21.

Fibronectin (FN), a glycoprotein encoded by the *FN1* gene, is involved in several biological processes, including cell adhesion, tissue development and wound healing 22. FN also has a role in TGFβ regulation 23. Moreover, FN expression increases under stimuli induced by TGFβ 24.

Moreover, the tenascins (TN), including TNR, TNC and TNX, are a highly conserved family of ECM glycoproteins. TNR is expressed only in the brain, whereas TNC and TNX are expressed in several organs and tissues, including in the joints 25. TNC has an important role in modulating the actions of TGFβ 26 and is also regulated by TGFβ 27. TNXB seems to regulate collagen synthesis or deposition 28. A recent study showed that TNX also regulates TGFβ bioavailability and modulates cell plasticity 29.

In the present study, we quantified the mRNA expression of the *TGFβ1, TGFβR1, LOX*, *PLOD1, PLOD2, COMP, FN1, TNC* and *TNXB* genes in glenohumeral synovium/capsule samples collected from patients with adhesive capsulitis and from controls. We also evaluated how these mRNA levels are associated with clinical features.

## MATERIALS AND METHODS

### Patients

The current study used a case-control study design (level 3 evidence). All patients and controls were treated at the Hospital São Paulo of the Universidade Federal de São Paulo. Each patient agreed to participate by signing a written consent form before data and sample collection. This study was approved by the ethics committee of the Universidade Federal de São Paulo (approval number: CEP 1918/11).

The case group was composed of 9 patients with idiopathic adhesive capsulitis of the shoulder in freezing or frozen stages who were diagnosed by clinical evaluation. During the clinical evaluations, the patients presented with pain, loss of motion and severe limitations during daily activities; no history of trauma or previous shoulder pathologies; and functional restriction of both active and passive shoulder motion. Magnetic resonance imaging (MRI) was used to exclude secondary stiff shoulder. The patients underwent arthroscopic shoulder capsular release after a concerted effort was made to treat them with conservative management for at least 5 complete months. In all cases, the physiotherapy had failed. Additionally,patients meeting the following exclusion criteria were omitted: generalized arthritis; previous compromise of the shoulder, such as major trauma, fracture, rotator cuff tear, calcifying tendinitis, or shoulder instability; and superior labral anterior and posterior (SLAP) lesions. Additionally, patients who did not agree with the informed consent terms were excluded. All enrolled patients underwent an arthroscopic procedure.

The control group consisted of 8 physically active subjects who underwent arthroscopically assisted treatment for acute acromioclavicular dislocation. None of the controls presented with a history of adhesive capsulitis. Moreover, radiological indication of glenohumeral capsule alteration was detected. A standard complete joint evaluation by arthroscopy confirmed that the controls did not present any other concomitant pathology in the shoulder.

All patients answered a preoperative questionnaire concerning gender, age at surgery, age of pain onset, duration of symptoms, bilaterality, suprascapular nerve block, physical activity, type of work and smoking habits (Table 1).

### Tissue samples

Tissue samples of approximately 2 mm^3^ were obtained from the anterior-inferior portion of the glenohumeral capsule during the arthroscopic procedure. To reduce sampling variation, only two of the authors (CC and BE) were responsible for collecting the tissue samples. The samples were collected as previously described 30-32. As the synovium is adhered to the capsule, it cannot be separated from the capsule using arthroscopic instruments.

To provide immediate stabilization of RNA, all synovium/capsule specimens were instantly preserved in Allprotect Tissue Reagent^®^ (Qiagen, Germany) and then stored at -20 °C.

### RNA extraction

An AllPrep DNA/RNA/miRNA Universal Kit (Qiagen, Germany) was used to purify total RNA from the synovium/capsule specimens. Tissue Lyser LT equipment (Qiagen, USA) was used to mechanically lyse the tissue samples. A NanoDrop ND-1000 spectrophotometer (Thermo Scientific, USA) was used to determine RNA concentration and quality. RNA integrity was verified by 1% agarose gel electrophoresis. The RNA samples were stored at -80 °C.

### mRNA expression analysis

Reverse transcription-quantitative polymerase chain reaction (RT-qPCR) was used to evaluate mRNA expression. First, a High-Capacity cDNA Archive kit (Life Technologies, USA) was used for cDNA synthesis. Then, RT-qPCR was performed as previously described using a ViiA 7 Real-Time PCR System (Life Technologies, USA) 30. To exclude technical variations, target and reference genes (Table 2) were analyzed on the same TaqMan Low-Density Array (TLDA) cards (Life Technologies, USA). All qPCR assays were performed in triplicate. The *B2M* and *HPRT1* genes were used as reference genes to normalize sample input amount. These genes were chosen based on a previous study of suitable internal controls for the evaluation of mRNA expression in shoulder capsule samples 33.

The relative cycle threshold method (Crt method) was used to quantify mRNA expression. In this method, the lower the cycle threshold value (Crt) value, the greater the number of initial target copies in the sample. Thus, low Crt values indicate high gene expression. The expression of the target genes was determined using the following equation: ΔCrt=target gene Crt – the mean of the reference genes Crt.

### Statistical analysis

All ΔCrt values are shown as the median with the interquartile range (IQR).

The gender and age distributions between the patients with adhesive capsulitis and the controls were compared using the Chi-square test and the Mann-Whitney test, respectively. The Mann-Whitney test was also applied to compare mRNA levels between the cases and the controls, as well as to investigate the possible associations between mRNA expression and preoperative clinical variables, such as gender, practice of sports involving the upper limbs, type of job (manual *versus* non-manual job) and smoking habits. Spearman’s correlation was used to assess possible correlations between mRNA levels and duration of symptoms or age at surgery: a value below 0.40 was considered a weak correlation, a value between 0.40 and 0.59 was considered a moderate correlation, a value between 0.6 and 0.79 was considered a strong correlation and a value ≥0.80 was considered a very strong correlation. For all analyses, a *p*-value<0.05 was considered statistically significant. Statistical analyses were performed using PASW (SPSS) software, version 18 (SPSS Inc., Chicago, USA).

## RESULTS

### Differences between cases and controls

Table 1 presents the clinical variables of the study participants. In the control group, 1 (12.5%) individual was female and 7 (87.5%) were males. The median age at the time of surgery was 31.44 years (IQR=13.5). The gender distribution did not differ between the study groups (*p*=0.05). However, the controls were significantly younger than the patients with adhesive capsulitis (*p*=0.012, Mann-Whitney test).

**Figure 1:**
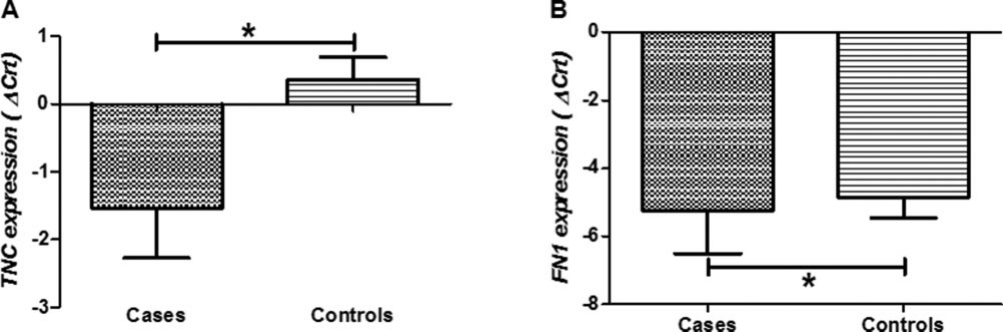
*TNC* (A) and *FN1* (B) expression levels in capsule samples collected from patients with adhesive capsulitis and controls. A lower delta cycle threshold value (ΔCrt) indicates higher gene expression. Box plots show the median and interquartile range. *A significant difference between groups by Mann-Whitney test (*p* <0.05).

Table 3 shows the median and IQR values for the expression levels of the studied genes in the samples from the patients and the controls. The patients with adhesive capsulitis had higher levels of *TNC* (*p*=0.005; Figure 1A) and *FN1* (*p*=0.043; Figure 1B) expression compared to the controls. No other significant differences were observed between the patients and the controls (*p*>0.05).

### Associations between the clinical characteristics of adhesive capsulitis and mRNA expression

**Figure 2:**
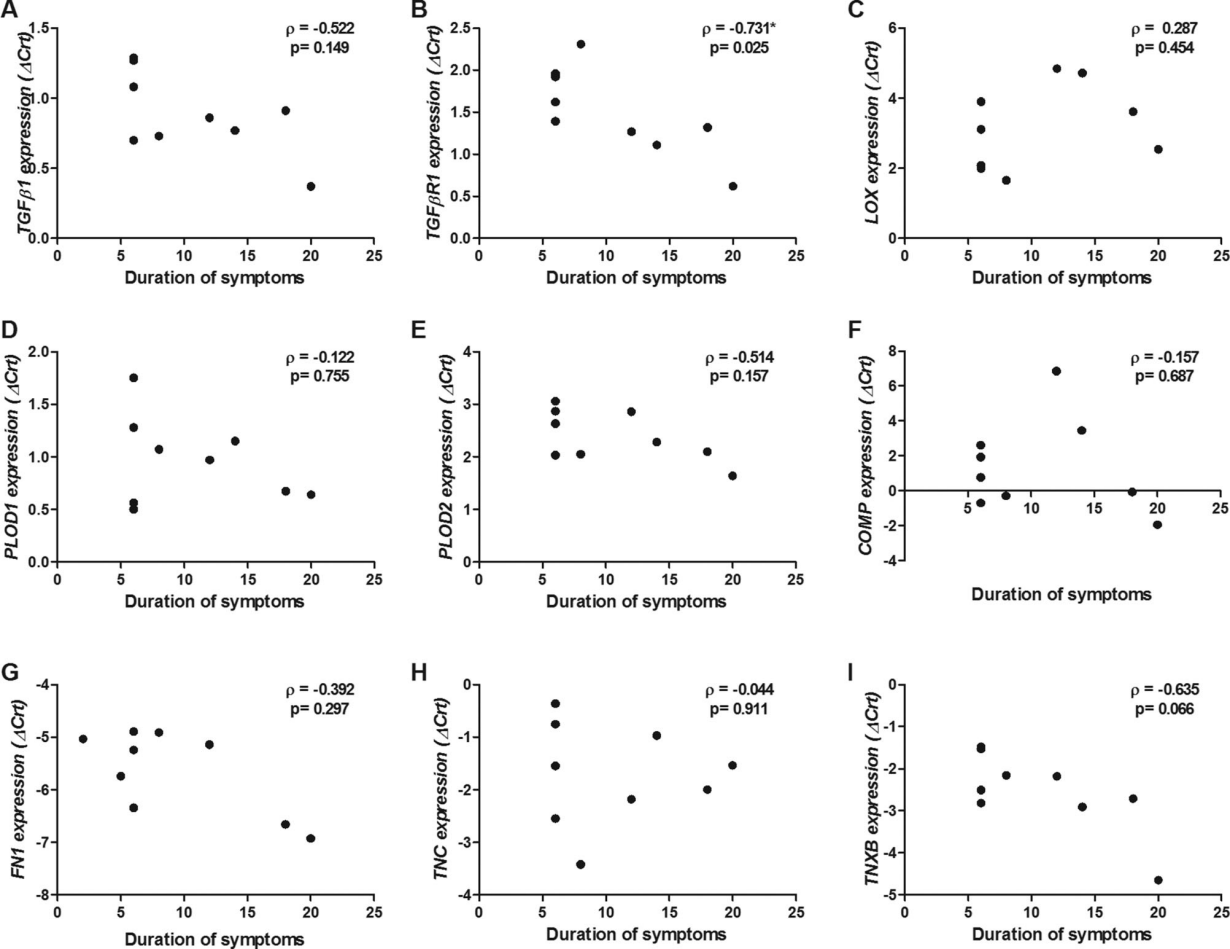
Correlation between gene expression and duration of adhesive capsulitis (months). A) *TGFβ1*; B) *TGFβR1*; C) *LOX*; D) *PLOD1*; E) *PLOD2*; F) *COMP*; G) *FN1*; H) *TNC*; and I) *TNXB.* ρ: Spearman correlation coefficient ("rho"). *Significant correlation (*p*<0.05). A lower delta cycle threshold value (ΔCrt) indicates higher gene expression.

Figure 2 shows the correlations between the duration of adhesive capsulitis symptoms and the expression levels of the studied genes. In the tissue samples, the expression of *TGFβR1* mRNA was significantly and directly correlated with the duration of symptoms (ρ=-0.731, *p*=0.025; Figure 2B).

No correlation was found between the age of the patients at the time of surgery and the age at symptom onset (*p*>0.05). Additionally, no association was found between the mRNA levels of the studied genes and any of the clinical features assessed in the patients with adhesive capsulitis (*p*>0.05).

## DISCUSSION

Although adhesive capsulitis is considered a self-limited disease, some patients show little to no improvement, maintain a limited range of motion and continue to experience shoulder pain. Non-operative treatment is the initial approach used for adhesive capsulitis. However, operative treatment (such as arthroscopic capsular release) may be considered when patients remain in pain and do not regain satisfactory range of motion after prolonged nonoperative treatment 34,35.

In this study, we found higher levels of *TNC* and *FN1* expression in glenohumeral synovium/capsule samples collected from patients with adhesive capsulitis compared to those collected from controls. TNC immunoreactivity was previously reported in other shoulder diseases, including in rotator cuff tendon tears and in the subacromial bursa of patients with impingement syndrome 36,37. In addition, we have previously found that both *TNC* and *FN1* mRNA levels were upregulated in the glenohumeral capsules of patients with traumatic anterior shoulder instability (unpublished data). TNC is a large hexameric ECM glycoprotein that has roles in cell adhesion, fibroblast migration and other processes related to tissue remodeling and wound healing 38,39. TNC is specifically expressed following tissue damage, being upregulated within 24 h of injury 38. It is activated after local injury and down-regulated after tissue repair or scarring is completed 40. Persistent expression of TNC is associated with several fibrotic diseases and with chronic non-healing wounds 38.

Therefore, we hypothesize that increased TNC expression may be a marker of capsule injury and the genes involved may participate in inflammatory and fibrotic processes in the glenohumeral capsule.

FN is essential for collagen fibril assembly 41. During the early phase of wound healing, FN is deposited at sites of injury and can induce inflammation; increase ECM deposition, including of FN and collagen; and activate fibroblasts. These pathways can create a vicious cycle that eventually induces keloid formation or fibrosis 42. Additionally, FN has been previously associated with Dupuytren's contracture 24. In this disease, FN can be found in its oncofetal form 43,44. Additionally, upregulation of FN1 has been associated with fibrosis in inflammatory orbital diseases 45, hepatic fibrosis 46, idiopathic pulmonary fibrosis 47,48 and liver fibrosis 49. These relationships indicate that this molecule may also be involved in the pathogenesis of other fibrosing diseases. Interestingly, Altrock et al. showed that blocking FN deposition using an FN assembly inhibitor (pUR4) resulted in decreased collagen accumulation and improved liver function during liver fibrogenesis 50. Although only a slight increase in FN1 expression was detected in the glenohumeral capsules of the patients with adhesive capsulitis in the current study, our results suggest that FN1 may play a role in the fibrotic process. Further investigation is still necessary to understand the dynamic transcriptional regulation of FN1 that occurs within the shoulder capsule.

We also observed that the expression of *TGFβR1* mRNA in the capsule was directly correlated with symptom duration in the patients with adhesive capsulitis. To the best of our knowledge, only one previous study has evaluated the role of the TGFβ receptor in adhesive capsulitis 5. Rodeo et al. analyzed both *TGFβ* and its receptor in capsule and synovium samples collected from patients with adhesive capsulitis and in those collected from controls 5. They performed a semi-quantitative analysis by comparing the frequency of positive staining between the groups. The authors described that the synovial and capsular cells of patients with adhesive capsulitis and synovitis showed clear TGFβ and TGFβR staining, whereas no or minimal staining was observed in the normal tissue specimens. The blood vessels of the affected tissues also presented staining for both proteins. Moreover, there was a higher frequency of positive TGFβ and TGFβR staining in the synovial cells of the patients with adhesive capsulitis. In addition, there was a greater frequency of positive TGFβ staining in the ECMs of patients with adhesive capsulitis compared to the controls, particularly in the capsule tissue.

In the present study, we found no differences in the expression of *TGFβ1* and *TGFβR1* mRNA between the cases and the controls. Because the synovium is adhered to the capsule and cannot be separated from the capsule using arthroscopic instruments, our investigation did not discriminate between gene expression in synovial and capsular tissues. However, molecular alterations in both tissues are important in adhesive capsulitis 51. In addition, our study is the first to use a quantitative approach to evaluate the role of *TGFβR1* in adhesive capsulitis. Our results suggest that *TGFβR1* may have a role in adhesive capsulitis, especially in the long-term disease.

To the best of our knowledge, this study is the first to quantify *TGFβ1*, *TGFβR1, LOX, PLOD1*, *PLOD2, COMP, FN1, TNC* and *TNXB* mRNA expression in the shoulder capsules of patients with adhesive capsulitis. However, this study has some limitations. First, few patients with adhesive capsulitis are surgically treated; as such, there is a limited number of tissue samples available for studies of gene expression 5,35,52-59. Therefore, some of our statistical analyses had reduced power to detect significant differences between the studied groups and false-negative results may have occurred. Second, we included patients who failed in conservative treatment in different phases of frozen shoulder, some in freezing and others in frozen stages. This heterogeneity may have also contributed to false negatives. Third, molecular alterations may occur in other capsule regions and may have a different etiological role in capsular injury 30-32. We evaluated the AI region because this portion of the capsule presented macroscopic injuries (i.e., a high level of inflammation) during arthroscopic examination of the studied patients. Although we did not detect a correlation between age and gene expression in the tissue samples collected from the patients with adhesive capsulitis, it is important to highlight that the age distribution between the patients and the controls was different. Thus, we cannot exclude that age might have influenced our findings. Finally, additional analysis of the protein products of the studied genes may be interesting because protein function is also affected by post-transcriptional and post-translational regulation.

Elevated expression of *TNC* and *FN1* mRNA may be a marker of capsule injury and may be involved in capsule inflammation and fibrosis. Upregulation of *TGFβR1* seems to be related to symptom duration in adhesive capsulitis; therefore, TGFβ signaling may play a role in this condition.

## AUTHOR CONTRIBUTIONS

Cohen C, Leal MF, Smith MC, Ejnisman B and Faloppa F conceived and designed the experiments. Cohen C, Belangero PS, Figueiredo EA, Andreoli CV, Pochini AC and Ejnisman B were involved in data collection. Cohen C, Belangero PS, Figueiredo EA, Figueiredo EA, Pochini AC and Ejnisman B were responsible for sample collection. Leal MF was involved in the genetic analysis. Cohen C, Leal MF, Belangero PS and Figueiredo EA performed the literature search. Leal MF and Smith MC were involved in data and statistical analyses. Cohen C and Leal MF wrote the first draft of the manuscript. All authors listed have contributed to all subsequent drafts and all have approved the final manuscript.

## Figures and Tables

**Table 1:** Distribution of clinical variables for patients with adhesive capsulitis.

Variable	Distribution
Age at surgery, years [median (IQR)]	51.7 (16.5)
Age at symptom onset, years [median (IQR)]	50.4 (14.5)
Gender [N(%)]	
Male	3 (33)
Female	6 (67)
Duration of condition, months [median (IQR)]	10.67 (10)
Bilaterality [N(%)]	
No	6 (67)
Yes	3 (33)
Practice of sports involving the upper limbs [N (%)]	
No	8 (89)
Yes	1 (11)
Type of job [N(%)]	
Non-manual	6 (67)
Manual	3 (33)
Smoking habits [N(%)]	
Non-smoker	8 (89)
Smoker	1 (11)

N: number of patients; IQR: interquartile range.

**Table 2:** Summary of reference gene and target gene assays.

Gene symbol	Name	Assay*
*TGFβ1*	Transforming growth factor, beta 1	Hs00998133_m1
*TGFβR1*	Transforming growth factor, beta receptor 1	Hs00610320_m1
*LOX*	Lysyl oxidase	Hs00942480_m1
*PLOD1*	Lysyl hydroxylases 1	Hs00609368_m1
*PLOD2*	Lysyl hydroxylases 2	Hs01118190_m1
*COMP*	Cartilage oligomeric matrix protein	Hs00164359_m1
*FN1*	Fibronectin 1	Hs00365052_m1
*TNC*	Tenascin C	Hs01115665_m1
*TNXB*	Tenascin XB	Hs00372889_g1
B2M**	Beta-2-microglobulin	Hs00984230_m1
HPRT1**	Hypoxanthine phosphoribosyl-transferase	Hs02800695_m1

* TaqMan probes were purchased as assay-on-demand products for gene expression (Life Technologies, USA).

** Reference genes for target gene expression normalization.

**Table 3:** Gene expression patterns in the glenohumeral capsules of patients with adhesive capsulitis and in controls.

Gene	Cases [ΔCrt; Median (IQR)]	Controls [ΔCrt; Median (IQR)]	*p*-value
*TGFβ1*	0.99 (0.62)	0.57 (0.52)	0.149
*TGFβR1*	1.77 (1.04)	1.47 (0.73)	0.923
*LOX*	3.16 (2.27)	3.37 (1.52)	0.700
*PLOD1*	0.95 (0.62)	1.07 (0.48)	0.336
*PLOD2*	2.39 (0.82)	3.11 (1.08)	0.068
*COMP*	1.39 (3.53)	-0.01 (2.05)	0.336
*FN1*	-5.65 (1.53)	-4.85 (0.79)	0.043*
*TNC*	-1.48 (1.72)	0.36 (1.31)	0.005*
*TNXB*	-2.55 (1.02)	-2.79 (1.03)	0.290

* Significant difference between groups by Mann-Whitney test (*p<*0.05). IQR: interquartile range. A lower delta cycle threshold value (ΔCrt) indicates higher gene expression.
